# FORM-TRACE: A formula-based method for tracking forest-management transitions at Harvard Forest from 1908 to 2026

**DOI:** 10.1016/j.mex.2026.104054

**Published:** 2026-07-18

**Authors:** Nophea Sasaki, Eain Dray Aung

**Affiliations:** Sasin School of Management, Chulalongkorn University, Bangkok, 10330, Thailand

**Keywords:** Corpus manifest, Keyword-domain matrix, PDF text extraction, Audit trail, Temporal aggregation, Documentary evidence

## Abstract

Long-term changes in forest management are documented across reports, plans, and scientific papers written for different purposes and with changing vocabularies. This makes it difficult to show how a documentary record was converted into a temporal claim. FORM-TRACE is a formula-based workflow that records corpus decisions, extraction quality, domain terms, and calculations before interpretation. We demonstrate it with Harvard Forest and New England documents dated 1908–2026. Of 257 PDFs inspected, 215 met the analytical criteria; 201 were extracted and scored, 14 were flagged as unreadable, scanned, or corrupted, and 42 methods-support references were kept outside the scored corpus. The method provides:

• A corpus manifest and extraction log that expose inclusion, exclusion, and coverage gaps;

• A keyword-domain matrix and five numbered equations that produce document- and period-level indicators;

• Saved score tables, plot data, and validation records that allow independent checking without redistributing copyrighted PDFs.

FORM-TRACE measures documented attention rather than management performance and keeps interpretation separate from scoring.

## Graphical abstract

**Figure:**
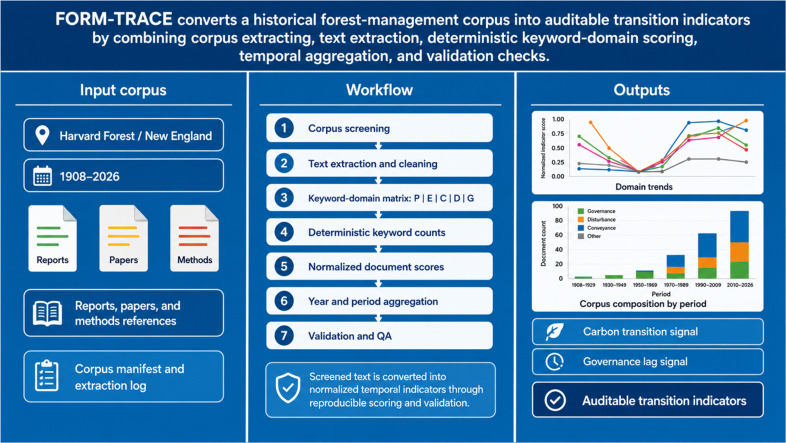

## Specifications table

**Table:** 

Subject area	Environmental science
**More specific subject area**	Formula-based documentary analysis; corpus audit trails; forest-management transitions
**Name of your method**	FORM-TRACE — Formula-based Open Reproducible Method for Transition and Corpus Evidence
**Name and reference of original method**	FORM-TRACE combines established practices in qualitative content analysis [1,2], text-as-data analysis [3,4], dictionary-based text analysis [5], and reproducible computational research [6,7] in a documented workflow for long-term forest-management corpora.
**Resource availability**	The FORM-TRACE reproducibility package has been deposited in Mendeley Data under reserved DOI 10.17632/7xnchr7bp3.1. The record will become publicly accessible after repository approval. The package contains the corpus manifest, extraction log, keyword-domain matrix, document-level and period-level scores, plot data, regenerated figures, file inventory, checksums, run report, and data dictionary. Source PDFs and full extracted text are not redistributed where copyright or licensing restrictions apply.

## Background

Forest-management change leaves a long and uneven documentary record. Annual reports, technical reports, field studies, policy documents, and scientific papers each capture only part of that record. They also differ in purpose, vocabulary [1,3]. Their layout and extractability also vary. Historical reports may describe harvest, regeneration, and yield, whereas recent papers may emphasize carbon exchange, disturbance, resilience, or governance. Some older PDFs are scanned images rather than searchable text. Before interpretation begins, we therefore need to know which files can be read, which require optical character recognition (OCR), and which should not enter the scored corpus.

The central problem is the path from documents to evidence. Narrative review and expert synthesis remain essential, but readers also need to see how the corpus was assembled, which files were retained, which were excluded, which documents failed extraction, which terms represented each domain, and how the indicators were calculated [6,[8], [9], [10]]. Without that record, a historical interpretation may be plausible while the evidence-processing decisions remain difficult to inspect.

This problem is especially important in forest-management research because management language changes over time. Older documents often describe production and silvicultural practice; later documents increasingly discuss ecosystem structure, carbon budgets, disturbance, climate risk, forest health, and implementation. A reproducible method must preserve historical terminology while making newer signals measurable. It must also keep analytical documents separate from methods-support references and unreadable files so that the results are not shaped by hidden exclusions.

FORM-TRACE was developed to make this document-to-indicator process explicit. It combines the discipline of qualitative content analysis with deterministic text analysis [[1], [2], [3],^11^]. The method records the corpus, logs extraction quality, defines a keyword-domain matrix, applies fixed equations, aggregates the results by year or period, and stores the figure data used for interpretation. These outputs do not replace expert judgement. They provide a documented basis for checking how textual attention changes across the corpus.

Harvard Forest in New England provides a suitable worked example because its documentary record spans historical forestry, ecological structure, carbon science, disturbance research, and governance-oriented management. The example is used to demonstrate the method, not to offer a complete history of Harvard Forest. The purpose of this MethodsX article is narrower: to explain the workflow in enough detail for another researcher to inspect, repeat, and adapt it.

## Method details

### Overview of the workflow

**Fig. 1:**
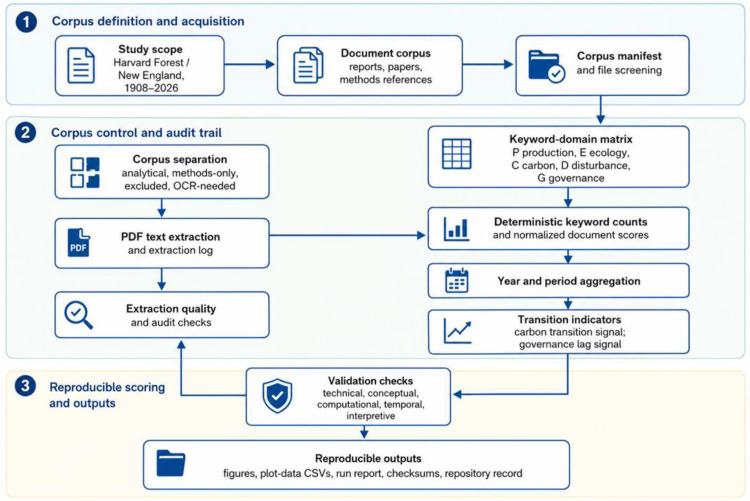
FORM-TRACE study workflow. The figure connects the analytical boundary, text extraction, deterministic scoring, aggregation, validation, and stored outputs. Each operation leaves a file or table that can be inspected before interpretation.

FORM-TRACE begins with a set of documents and a question about change over time. The method does not ask a model to infer that change. We record the corpus, define the terms that represent each domain, apply fixed equations, and retain the intermediate files. Fig. 1 summarizes the eight operations described below:

1. Define the transition question, geographic scope, time period, source types, and analytical domains.
2. Build a corpus manifest and classify each file as analytical, methods-support, duplicate, excluded, corrupted, or requiring optical character recognition (OCR).
3. Extract text from analytical PDFs and record readability, word count, and failure flags [6,7,10].
4. Construct and review the keyword-domain matrix before scoring begins.
5. Count domain terms deterministically and normalize the counts by document length.
6. Calculate the two composite transition signals.
7. Aggregate document scores by year and historical period, retaining the number of documents in each period.
8. Validate the inputs and outputs, then generate figures and reproducibility records from saved tables.

### Required inputs

FORM-TRACE requires three inputs: a document corpus, a reproducible working environment, and decisions about scope and domains made before scoring. The method is formula-based rather than a standalone software package. It can be implemented in Python, R, spreadsheet software, or another reproducible tabular environment, provided that the corpus manifest, keyword matrix, equations, and validation rules are preserved.

In the worked example, Python was used for PDF extraction, keyword counting, aggregation, and figure preparation. The repository run report and data dictionary record the file structure, output columns, and calculation rules used in the analysis.

1. Document corpus: historical annual reports, peer-reviewed papers, technical reports, management plans, policy documents, archival files, and methods-support references.
2. Working environment: software capable of PDF text extraction, keyword and phrase counting, tabular aggregation, and figure generation.
3. Analytical definitions: research question, geography, time period, source types, domain labels, keyword terms, exclusion rules, period bins, and validation checks.

### Recommended folder structure

Before any score is calculated, the project files are organized so that each decision leaves a visible record. The folders below are the storage layer of the method. They keep analytical documents, methods-support references, extraction records, scores, figures, and run logs separate.

The worked example used the following folders:

1. References/papers: core peer-reviewed papers and target publications.
2. References/trending: chronologically sampled forest-management literature.
3. References/reports: historical reports, manifests, and download logs.
4. methods_references: text-extraction, content-analysis, and methodological support references that justify the workflow but are excluded from forest-management scoring.
5. FORM-TRACE-project/01_corpus_manifest: corpus manifest and document inventory.
6. FORM-TRACE-project/02_text_extraction: extraction logs and extracted text records.
7. FORM-TRACE-project/03_keyword_matrix: domain terms, term variants, and coding notes.
8. FORM-TRACE-project/04_domain_scores: document-level, year-level, and period-level score tables.
9. FORM-TRACE-project/05_figures: figures generated from saved output tables.
10. FORM-TRACE-project/06_logs: run reports and reproducibility logs.

The eight steps below explain how these folders are populated. The sequence matters: the transition is defined first; the corpus boundary is fixed second; readable text is then extracted; and only after those decisions are recorded are terms counted and indicators calculated.

**Step 1: *Define the transition***

We first define the transition that the corpus is expected to reveal. This decision comes before keyword selection or scoring because the terms counted later must be linked to a stated research question. In the worked example, the transition concerns a shift in documented attention from production-oriented management toward ecological structure, carbon and ecosystem function, disturbance and climate risk, and governance implementation.

The worked-example transition is expressed as: timber production → ecological structure → carbon function → disturbance risk → governance implementation.

The output of Step 1 is TRANSITION_DEFINITION.md. It records the research question, geographic scope, time period, source types, analytical domains, exclusion rules, and limits of interpretation. The keyword-domain matrix in Step 4 is derived from this definition.

**Step 2: *Build and separate the document corpus***

We then build the corpus manifest and assign every file to a corpus group before scoring begins [6,7,12]. A document enters the analytical corpus only when it is relevant to the transition question and readable enough for text extraction. Methods-support references justify the workflow but are not scored as evidence of forest-management discourse. Duplicates, excluded files, corrupted files, and OCR-needed files remain visible in the manifest. This prevents the final corpus from being created by silent deletion.

For each file, the manifest records: record_id: a unique document identifier; filename and folder: the source filename and its storage location; title_inferred, author_inferred, and year_inferred: bibliographic metadata obtained from the file or document text when available; source_type: article, report, annual report, methods reference, policy document, or another source type; corpus_group: analytical, methods-support, excluded, duplicate, corrupted, or OCR-needed; pdf_readable, text_extracted, and word_count: whether the file was readable, whether text was obtained, and how many words were available; notes: a plain-language explanation of ambiguity, duplication, OCR needs, extraction failure, or another file-level decision.

Together, Steps 1 and 2 define the analytical boundary of FORM-TRACE. Only documents classified as analytical and successfully extracted proceed to the scoring workflow.

**Step 3: *Extract text and log quality***

We process every analytical PDF with the same extraction routine. For each file, the extraction log records whether text was obtained, the character and word counts, whether the file is image-only or contains little extractable text, and any error message. Image-only files are marked for optical character recognition (OCR) and are not scored until readable text is available. Failed cases remain in the log rather than being silently removed.

The extraction success rate is calculated as:

(1) $$ESR=\frac{Nn_{scored}}{N_{analytical}}\times 100$$

where ESR is the extraction success rate; N_scored is the number of analytical documents successfully extracted and scored; and N_analytical is the number of documents assigned to the analytical corpus. In the worked example, ESR = (201 / 215) × 100 = 93.49%.

**Step 4: *Construct and review the keyword-domain matrix***

The keyword-domain matrix links the qualitative transition definition to the numerical scores [1,3,5]. We compiled candidate terms from the domain definitions and checked them against sample documents from different periods. Multiword phrases were preferred when a single word produced ambiguous matches. Terms that generated false positives were refined, restricted by an exclusion note, or removed [4,13]. Matching is case-insensitive unless KEYWORD_MATRIX.csv states otherwise.

Keyword-domain matrix

The matrix uses five domain labels. Each label connects a forest-management concept to the terms counted for that concept:

P, production and silviculture: timber, harvest, yield, rotation, regeneration, stocking, sawtimber, thinning, logging.

E, forest composition and ecological structure: mixed hardwoods, hemlock, white pine, succession, old growth, biodiversity, habitat, canopy structure.

C, carbon and ecosystem function: carbon storage, carbon sequestration, biomass, soil carbon, CO2 exchange, net ecosystem exchange, phenology.

D, disturbance, climate, and forest health: hurricane, windthrow, ice storm, drought, warming, climate change, resilience, pests, mortality.

G, governance and implementation: private woodlots, family forests, conservation easements, forest carbon protocol, carbon markets, permanence, policy.

**Step 5: *Score documents deterministically***

FORM-TRACE uses deterministic keyword-domain matching rather than generative-AI classification [1,3,5]. For each document d and domain k, the method counts the terms listed in KEYWORD_MATRIX.csv and normalizes the count by document length [14].

Document-domain scores are calculated as:

(2) $$S_{\left(d,k\right)}=100\times \frac{N_{\left(d,k\right)}}{W_{\left(d\right)}}$$

where S_(d,k)_ is the normalized score for document *d* and domain *k*; N_(d,k)_ is the number of matched domain terms in document *d* for domain k; and W_(d)_ is the total word count of document *d*.

Eq. (2) reports keyword density per 1000 words, allowing documents of different length to be compared.

The values are keyword-match frequencies, not bounded index scores. They therefore have no fixed maximum such as 10 or 100. Interpretation is based on the observed range in the corpus and on inspection of the underlying documents.

The highest normalized score identifies the dominant textual signal in a document. Secondary scores and document notes are retained because a short document can produce a high value from only a few repeated terms.

**Step 6: *Calculate transition indicators***

The worked example uses two composite indicators. The Carbon Transition Signal is calculated as:

(3) $$CTS_{\left(d\right)}=C_{\left(d\right)}+D_{\left(d\right)}+G_{\left(d\right)}-P_{\left(d\right)}$$

where CTS_(d)_ is the Carbon Transition Signal for document *d*; C_(d)_, D_(d)_, G_(d)_, and P_(d)_ are the normalized scores for carbon and ecosystem function, disturbance and climate risk, governance and implementation, and production and silviculture, respectively.

Eq. (3) is a textual contrast. Negative values indicate stronger production-oriented language; positive values indicate stronger carbon, disturbance, and governance-oriented language.

The Governance Lag Signal is calculated as:

(4) $$GLS_{\left(d\right)}=\left[C_{\left(d\right)}+D_{\left(d\right)}\right]-G_{\left(d\right)}$$

where GLS_(d)_ is the Governance Lag Signal for document *d*; C_(d)_ is the carbon and ecosystem-function score; D_(d)_ is the disturbance and climate-risk score; and G_(d)_ is the governance and implementation score.

Eq. (4) compares scientific and risk-related attention with governance and implementation attention. It is not a measure of policy effectiveness. Both composite signals are intended for comparison within this corpus; they should not be compared across studies unless the same domain definitions and keyword matrix are used.

**Step 7: *Aggregate by year and period***

Document-level scores are aggregated by year and by historical period. Period aggregation is useful because the corpus is unevenly distributed over time. The worked-example periods are 1908–1930, 1931–1950, 1951–1970, 1971–1990, 1991–2010, 2011–2020, and 2021–2026.

For each period p and domain k, the period score is calculated as:

(5) $${\bar{S}}_{p,k}=\frac{1}{n_{p}}\sum_{d\in D_{p}}S_{d,k}$$

for all scored documents *d* in period *p*. where S̄_(p,k)_ is the mean score for period *p* and domain *k; n_p* is the number of scored documents in period *p*; and S_(d,k)_ is the document-domain score from Eq. (2).

Eq. (5) makes the period summaries comparable while keeping the number of supporting documents visible.

After screening, no eligible document dated 1951–1970 was present in the assembled corpus. We retained the empty period to make this coverage gap visible. It may reflect archive availability or digitization rather than an absence of forest management or forest-management discourse; consequently, the period is reported as *n* = 0 and is not assigned a substantive zero.

**Step 8: *Generate reproducible figures and reports***

**Fig. 2:**
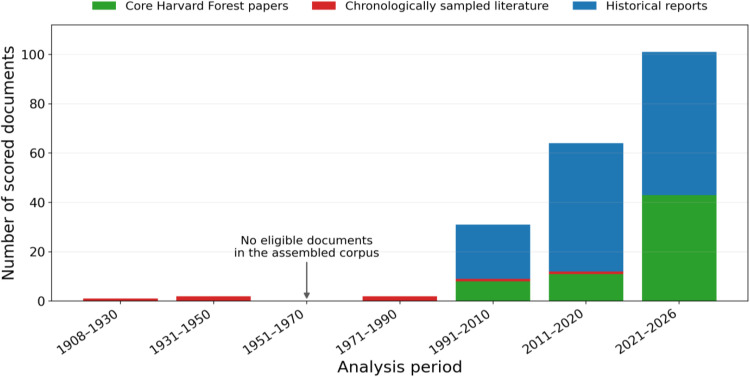
Corpus composition and document density by period. The 1951–1970 interval contains no eligible documents in the assembled analytical corpus. The empty interval is retained as a coverage gap and must not be interpreted as an absence of forest-management activity or discourse.

Fig. 2 reports the number of scored documents in each period and corpus group. Its purpose is methodological: it shows where the evidence base is dense, where it is sparse, and why low-density periods require cautious interpretation.

**Fig. 3:**
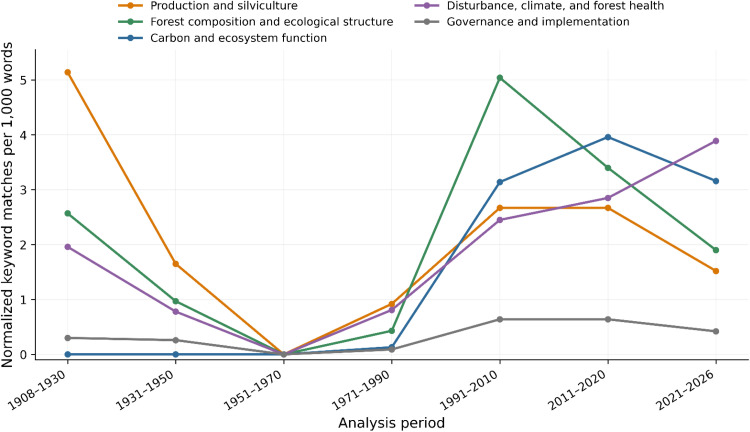
Documented attention across five forest-management domains. Values are average normalized keyword-match counts per 1000 words. The legend is placed above the plotting area, and sparse early periods should be read as limited historical baselines.

Fig. 3 shows how the five domains vary across time after normalization. The lines represent average keyword matches per 1000 words. They describe changing textual attention in the scored corpus, not ecological condition or management performance.

**Fig. 4:**
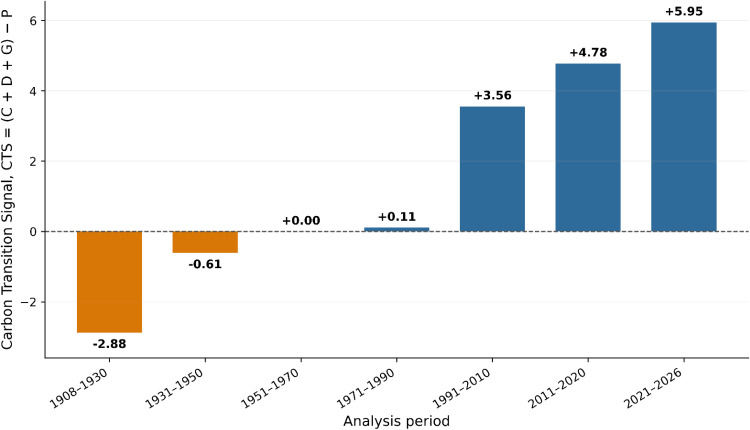
Carbon Transition Signal by period. Positive values indicate stronger carbon, disturbance, and governance language relative to production language.

Fig. 4 shows the Carbon Transition Signal, calculated with Eq. (3). It summarizes the relative movement from production-oriented language toward carbon, disturbance, and governance language.

**Fig. 5:**
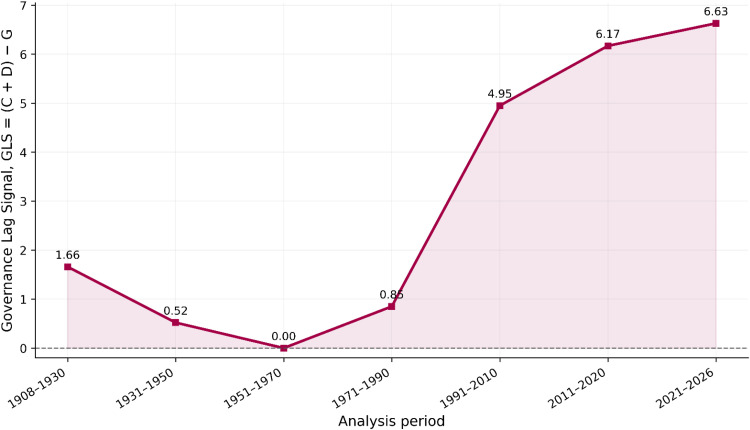
Governance Lag Signal by period. Higher values indicate that carbon and disturbance language exceeds governance and implementation language.

Fig. 5 shows the Governance Lag Signal, calculated with Eq. (4). It compares the prominence of carbon and disturbance language with governance and implementation language. Higher values mean that biophysical and risk-related discourse is more visible than governance language in the scored corpus.

**Fig. 6:**
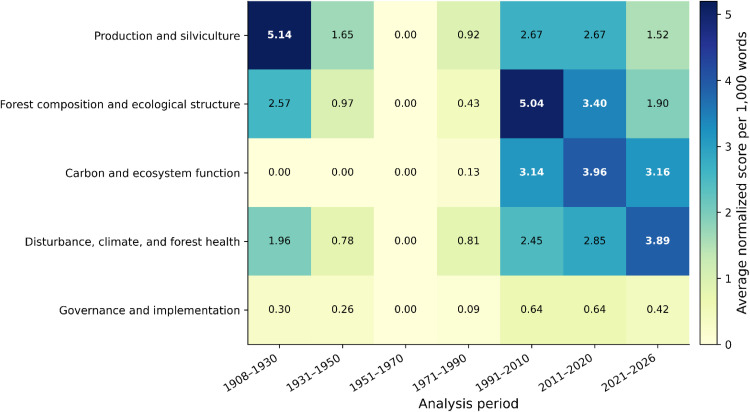
Attention heatmap of forest-management domains across periods. Values are average normalized keyword-match counts per 1000 words.

Fig. 6 presents the same five domains as a heatmap. This view allows readers to compare periods and domains at once and identify where textual signals are concentrated or weak.

All figures were regenerated from saved output tables. Larger labels and non-overlapping legends were used, and the corresponding plot-data CSV files are included in the data package.

## Method validation

FORM-TRACE is validated by making each transformation inspectable. The method does not treat keyword scores as direct evidence of field-level management change. Readers can inspect the corpus decisions, extraction record, domain terms, equations, score tables, period counts, and interpretation limits [6,8]. Human review remains part of reliability checking and interpretation [7,9,15].

### Validation layers

The validation checks are organized as follows:

Technical validation: confirm file identifiers, extraction status, character and word counts, OCR flags, duplicate records, missing years, and failed cases; the manifest and extraction log preserve these checks.

Conceptual validation: review domain definitions, candidate terms, ambiguous matches, exclusions, and the separation of methods-support references from analytical documents; the transition definition and keyword matrix preserve these decisions.

Computational validation: rerun the fixed equations from saved tables and check the document totals and period summaries; the document-domain and period-level score tables preserve the results.

Temporal validation: report the number of scored documents in every period and retain empty periods as coverage gaps rather than substantive zeroes.

Interpretive validation: inspect the underlying documents before making historical claims and state explicitly that textual attention is not the same as field-level practice.

### Worked-example efficiency

In the Harvard Forest in the New England worked example, FORM-TRACE converted a large and uneven document set into auditable outputs. The workflow inspected 257 PDF files, retained 215 documents in the analytical corpus, extracted and scored 201 documents, flagged 14 failed or OCR-needed documents, separated 42 methods-support references, and generated figure data from saved score tables.

### Efficiency comparison

The efficiency comparison is summarized as follows:

Corpus inventory: a conventional manual approach can be slow, inconsistent, and difficult to audit; FORM-TRACE uses a manifest-based inventory with document IDs and corpus groups.

PDF extraction: failures are often undocumented in manual review; FORM-TRACE logs extraction status, word counts, OCR flags, and errors.

Coding: narrative notes and implicit judgement can be difficult to reproduce; FORM-TRACE uses an explicit keyword-domain matrix with reviewable terms.

Scoring: manual scoring is difficult to reproduce across documents; FORM-TRACE uses deterministic normalized scores from saved scripts and tables.

Failed documents: manual workflows may exclude failed files without a visible trail; FORM-TRACE keeps failed, scanned, or corrupted documents cataloged.

Reuse: a conventional review can be hard to transfer to another transition problem; FORM-TRACE can be reused by replacing the corpus, domains, periods, and indicators.

## Limitations

Keyword-domain scoring measures textual attention, not forest-management practice. A document may discuss carbon, disturbance, or governance without those ideas being implemented on the ground [3,4].

The corpus is uneven over time. Evidence before 1990 is sparse and depends heavily on historical reports, while later periods contain more peer-reviewed literature. Period-level scores must therefore be interpreted together with document counts and the coverage gap in 1951–1970.

PDF quality affects reproducibility. Scanned reports, image-only documents, corrupted files, and complex layouts may require optical character recognition or manual checking. FORM-TRACE does not remove this problem; it records it.

The keyword matrix can miss older terminology, synonyms, and context-specific language. Domain assignment therefore requires expert review and iterative refinement [4,13].

Governance language may occur outside scientific corpora, especially in policy documents, management plans, legal records, and institutional reports. A weak governance signal in the scored corpus should not automatically be interpreted as weak governance practice.

Language-assistance tools were kept outside the empirical workflow. They did not define domains, classify documents, count terms, calculate scores or indicators, generate data, or determine interpretations. The empirical outputs were produced from documented formulas and saved tables.

## Ethics statements

This method article analyzes publicly available documents and does not report experiments involving humans or animals. Future applications involving human-participant data would require the relevant ethics review and consent procedures.

## Declaration of generative AI and AI-assisted technologies

During manuscript preparation, the authors used AI-assisted language tools for organization, drafting support, and editorial refinement. The authors defined the FORM-TRACE method, corpus structure, analytical domains, equations, and interpretation limits; checked the references and outputs; and approved the final manuscript. AI tools were not used to select the analytical corpus, classify documents, count terms, calculate scores or indicators, generate data, or make scientific interpretations.

## Funding

This research was funded by Sasin’s Internal Funding under the projects “RabbitESG and TreeTaishi”.

## CRediT authorship contribution statement

**Nophea Sasaki:** Conceptualization, Formal analysis, Funding acquisition, Investigation, Methodology, Project administration, Software, Visualization, Writing – original draft. **Eain Dray Aung:** Data curation, Validation, Visualization, Writing – review & editing.

## Declaration of competing interests

The authors declare no competing interests.

## Data Availability

Data will be made available on request.

## Appendix Supplementary materials

Image, application 1
