# Supplementary material for: FORM-TRACE: A formula-based method for tracking forest-management transitions at Harvard Forest from 1908 to 2026
Source: MethodsX. 2026 Jul 18;17:104054. doi: 10.1016/j.mex.2026.104054 (PMC13392954; doi:10.1016/j.mex.2026.104054)
Supplement: Supplementary file 1 [file mmc1.docx]

**Supplementary material and additional information**

***Data and code availability***

The FORM-TRACE repository is available in Mendeley Data: Sasaki, Nophea (2026), “FORM-TRACE: a reproducible workflow for converting forest-management documents into auditable transition indicators”, Mendeley Data, V1, doi: 10.17632/7xnchr7bp3.1. The repository contains the corpus manifest, extraction log, keyword-domain matrix, document-level scores, year-level and period-level scores, figure-specific plot data, generated figures, run report, file inventory, checksums, and data dictionary. Source PDFs are not redistributed where copyright or licensing restrictions apply. Where redistribution is not permitted, the repository provides document metadata, source URLs where available, extraction logs, scoring outputs, figure data, and reproducibility documentation sufficient for users to rerun the workflow with legally accessible copies.

***Recommended repository items***

Recommended repository item | Purpose

TRANSITION_DEFINITION.md | Defines the transition, scope, domains, and exclusion rules.

CORPUS_MANIFEST.csv | Lists all inspected documents and corpus groups.

EXTRACTION_LOG.csv | Records extraction status, word counts, OCR flags, and errors.

KEYWORD_MATRIX.csv | Stores terms, domains, variants, and notes.

DOCUMENT_DOMAIN_SCORES.csv | Stores normalized document-level domain scores.

DOMAIN_SCORES_BY_YEAR.csv / DOMAIN_SCORES_BY_PERIOD.csv | Stores temporal aggregations.

TRANSITION_FINDINGS.md and RUN_REPORT.md | Document transition findings, limitations, run metrics, and interpretation constraints.

Figure-generation documentation | Figure-generation notes and figure-specific plot-data CSV files that reproduce the displayed values in Figures S1.

***Supplementary material***

Supplementary material and reproducibility files are available through the Mendeley Data repository: Sasaki (2026), “FORM-TRACE: a reproducible workflow for converting forest-management documents into auditable transition indicators”, Mendeley Data, V1, doi: 10.17632/7xnchr7bp3.1. The repository includes the transition definition, corpus manifest, extraction log, keyword-domain matrix, document-level scores, year-level and period-level scores, figure-specific plot data, transition indicator formulas, run report, generated figures, data dictionary, file inventory, checksums, and reproducibility documentation. Source PDFs are not redistributed because of copyright and licensing restrictions.
